# Use of the Spectroscopy-Based Veggie Meter^®^ to Objectively Assess Fruit and Vegetable Intake in Low-Income Adults

**DOI:** 10.3390/nu13072270

**Published:** 2021-06-30

**Authors:** Jennifer Di Noia, Werner Gellermann

**Affiliations:** 1 Department of Sociology, William Paterson University, Wayne, NJ 07470, USA; 2 Longevity Link Corporation, Salt Lake City, UT 84108, USA; werner@longevitylinkcorporation.com

**Keywords:** fruit and vegetable intake, demographics, income, adults, Veggie Meter^TM^

## Abstract

Reflection spectroscopy is an emerging approach for noninvasively assessing dermal carotenoids as a biomarker of fruit and vegetable (FV) intake. This study sought to profile and identify determinants of scores from a reflection spectroscopy device (the Veggie Meter (VM)^®^) among 297 urban, primarily Hispanic low-income adults served by the Special Supplemental Nutrition Program for Women, Infants, and Children (WIC). The repeatability of the scores and bi- and multivariate relationships between VM scores, self-reported FV intake measured by a brief screener, and participant characteristics were examined. The mean VM score was 270 (range 0–695); 3- and 6-month test-retest correlations were positive and strong (r = 0.79 and 0.55, respectively). VM scores were negatively associated with body mass index (BMI; r = −0.22) and were higher among participants of Ecuadorian, Dominican, and Mexican Hispanic origin relative to those of Puerto Rican origin; foreign- vs. US-born participants, breastfeeding vs. non-breastfeeding participants, nonsmokers vs. smokers, and participants who consumed three or more cups of FV/day relative to those who consumed less than three cups of FV/day. Foreign-born nativity, consumption of three or more cups of FV/day, and smaller body size were determinants of increased VM scores. Although replication studies are needed to confirm these findings, investigators working with similar populations are encouraged to use the VM to longitudinally track FV intake and to target determinants of the scores in observational and intervention studies of FV intake as measured by the VM.

## 1. Introduction

Fruits and vegetables (FV) are the cornerstone of a healthy diet and are recommended for the prevention of disease [1,2]. Accurate assessment of FV intake is therefore essential for determining intake patterns, types and amounts of FV that are optimal for health, and evaluating FV interventions [3,4]. Yet, self-report approaches, i.e., 24-h recalls, food records, food frequency questionnaires, and FV screeners continue to be the mainstay of dietary assessment methods [5]. Self-report methods are prone to time burden, subjective biases, and intervention-related biases that can result in the misestimation of intake [6]. Other limitations include under- or over-reporting due to social bias and participants’ literacy [3]. Such limitations have motivated the development of reliable and objective biomarkers as indicators of dietary intake [7].

Carotenoids are a category of health-promoting phytonutrients found in a variety of FV [6,7]. Blood concentrations of carotenoids are considered the gold standard biomarker for FV intake [7]. Studies to date have relied on carotenoid analyses in plasma or serum by the use of high-performance liquid chromatography [7]. Although this approach is proven, key disadvantages are the cost of phlebotomy, sample processing/storage, sample analysis, and the necessity of venipuncture, which may introduce selection bias as some people are unwilling to give blood [7].

Skin carotenoid status is a noninvasive method of assessing FV intake that is becoming more available and acceptable for research use [3]. The Veggie Meter (VM; Longevity Link Inc., Salt Lake City, UT, USA) is a commercially available device that uses reflection spectroscopy to measure the level of carotenoid pigments in a person’s skin by scanning the tip of the finger. A score is produced which scales linearly with tissue carotenoid levels, with higher scores indicating higher carotenoid levels. Very few scores exceed a level of 800, which was established as the high end in a visual dashboard display of the levels. The device is compact and portable, easy to use without requiring intensive training, and results are immediate (scans can be taken in triplicate in less than 2 min) [3,8]. VM scores have good interindividual variation; are correlated with skin biopsy, blood carotenoid concentrations, and self-reported FV and carotenoid intake; and are repeatable, all necessary characteristics for a biomarker of nutritional status and food intake [8]. The VM is therefore ideal for observational and intervention studies of FV intake [3,6,8,9].

Data are increasingly available on VM scores in diverse adult samples (Table 1) [8,9,10,11,12,13,14,15,16,17,18,19,20,21]. Guidance on the interpretation of scores also has been published. For example, in a recent study, the scores differentiated adults who consumed three or more servings of FV/day as determined by two 24-h recalls from those consuming less than three servings/day [12]. Based on serum B carotene measurements, Rush at al. determined that a healthy VM score was between 280 and 480 [22]. Each 100 units of the VM score corresponds to approximately one serving (cup)/day of FV consumed.

Previous work has shown that VM scores are positively associated with nutrition knowledge, Asian ethnicity, weekly intakes of dark green leafy vegetables, carrots, and pumpkin; and intakes of carotenoid-rich FV, and negatively associated with weight and body mass index (BMI) [8,10,16,17]. The scores differed by receipt of federal nutrition assistance benefits (scores were higher among benefit recipients relative to non-recipients), lutein supplementation (scores were higher among adults who were supplementing with lutein relative to those who were not) and smoking status (scores were higher among nonsmokers than past and current smokers) [11,17]. Findings regarding whether the scores differ by sex are equivocal. In two studies, the scores were higher in men than women [11,12]. In a third study, the scores were higher in women than men, and in a fourth, the scores did not differ by sex [16,17]. Although these findings are informative, far less is known about VM scores in low-income samples and whether the scores differ among demographic subgroups as found elsewhere. 

In low-income adults, FV intake is associated with such characteristics as age, race, ethnicity, and nativity; language preference and length of time in the U.S. (among the foreign-born), pregnancy, breastfeeding, and food security status; educational attainment, and car ownership and access [23,24,25,26,27,28,29]. Similar associations with VM scores may be expected considering that variables associated with self-reported FV intake are also related to biomarkers of intake (potassium excretion and plasma vitamin C) [30]. Yet, studies relating a comprehensive set of demographic variables to VM scores are lacking. 

This study aimed to (1) profile VM scores among low-income adults served by the Special Supplemental Nutrition Program for Women, Infants, and Children (WIC), (2) assess the repeatability or test–retest reliability of skin carotenoid measurements, (3) examine bivariate relationships between VM scores, the aforementioned demographic characteristics, and self-reported FV intake, and (4) identify determinants of the scores. Findings will add to the limited data on VM scores in this population.

## 2. Materials and Methods

### 2.1. Design and Sample

This study was a secondary analysis of data from a recently completed dietary intervention study [31]. The setting was a large, WIC agency located in a densely populated, urban area of New Jersey, USA. The intervention was piloted with three of the agency’s 17 sites (one randomized to the intervention study group and two to the usual care control group) with 297 primarily Hispanic adults (160 enrolled at the intervention site and 137 at control sites). At baseline, participants were contacted via telephone prior to forthcoming appointments with WIC and were orally administered an outcome battery that included measures of demographics and self-reported FV intake. During appointments, biometric measures (height, weight, and carotenoid levels) were taken. Across sites, all participants were contacted to complete follow-up measures at mid- and post-intervention (3 and 6 months post-baseline, respectively). As at baseline, research staff administered the outcome battery to participants prior to appointments with WIC; during appointments, biometric measures were taken. The study was approved by the Institutional Review Board of William Paterson University (2018–339) and registered with ClinicalTrials.gov (NCT04038385). All participants provided informed consent prior to their study involvement (verbally, prior to completing telephone assessments and in writing, prior to completing in-person assessments). 

### 2.2. Measures

#### 2.2.1. Carotenoid Levels (VM Scores)

Dermal carotenoid levels were assessed with the VM by scanning the tip of the index finger. For a measurement, the finger was inserted into the instrument’s finger cradle. Via a laptop interface, the VM analyzed the light that was reflected from the fingertip to derive a carotenoid score. The device was operated in an averaging mode which produces an average score for three sequential measurements. The VM was calibrated (using dark and white calibration sticks provided by the manufacturer) each time it was turned on and every 1–2 h thereafter. 

Measurements were not affected by nail length or nail polish as the finger cradle is designed such that the top of the finger is positioned above the surface of the mold, ensuring there is ample room for long fingernails. Moreover, only the bottom tissue of the fingertip is measured, not the top, so nail length, polish, or other topical nail properties do not influence the results.

#### 2.2.2. Participant Characteristics and Self-Reported FV Intake

Participants reported their age in years. Those identifying as Hispanic were asked to report their origin (for those reporting more than one origin, origin was recorded as the first origin reported). Participants were also asked if they identified (yes/no) as any of the following races: Black or African American, White, Asian, American Indian or Alaska Native, and Native Hawaiian or Other Pacific Islander. Few participants (≤6%) reported more than one race or a race other than Black/African American and White. Consistent with previous research demonstrating that Hispanics adults consider their ethnicity to be their race, nearly all Hispanic participants (98%) did not identify as any race [32]. A combined race/ethnicity variable was therefore created (categories were Hispanic, non-Hispanic Black/African American, and non-Hispanic white or other). 

Nativity was assessed by asking participants if they were born in the US (yes/no) and if not, how many years they lived in the US and their preferred language (coded as English and other than English). As greater acculturation is associated with English language preference [26], the item on language preference was included as a measure of acculturation among foreign-born participants, with participants preferring to speak English considered more acculturated than those preferring to speak a language other than English.

Participants reported the highest year or grade they completed in school (coded as some high school or less, high school diploma or equivalent, and more than high school) and whether they were pregnant, breastfeeding, and owned a reliable car (yes/no). Participants also reported how often they could use the car (if owned) or how easy it would be to borrow a car (if not owned). Responses (on a 5-point scale) were combined to form a measure of car access (score range = 1 (poor) to 5 (excellent)). 

Food security status was assessed with an item from the US Department of Agriculture’s Household Food Security Survey Module (“Have you or other adults in your household worried whether your food would run out before you got money to buy more?”) [33]. As the interest was in assessing current food security status, the reference period was changed from the past 12 months to the past month. Participants also reported whether they smoked cigarettes (response options were not at all, some days, and every day (those reporting some days and every day were classified as smokers), were exposed to secondhand smoke in the past 7 days (yes/no) and were taking supplements (yes/no).

Weight status was measured as body mass index (BMI), calculated as weight in kilograms divided by height in square meters [34]. Height and weight were measured with participants wearing light clothing without shoes using standardized methods and equipment [35]. As weight and height were measured at time of study entry, among pregnant participants, pre-pregnancy BMI could not be determined. The recorded weights in this group were therefore adjusted by subtracting the number of pounds, on average, gained during pregnancy (using available data), and the adjusted weights were used to estimate pre-pregnancy BMI [36]. Participants were classified into the following weight status categories based on their BMI: underweight (BMI < 18.5), normal weight (BMI 18.5–24.9), overweight (BMI 25.0–29.9), and obese (BMI ≥ 30) [34]. Few participants (*n* = 12 or 4%) were underweight; therefore, the underweight and normal weight categories were combined. Participants also completed a 2-item measure of meeting physical activity guidelines shown to be reliable with validity similar to that of more detailed self-report activity measures [37]. Item responses were assigned point values and summed to derive a total score (used to classify participants as meeting physical activity guidelines [yes/no]).

Self-reported FV intake was assessed with a brief screener developed by the National Cancer Institute [38]. This 2-item measure queries the number of cups of FV (including 100% FV juices) the participant typically consumes per day [38]. To facilitate the estimation of food portions, participants were told that a cup was about the size of their fist [39]. The measure is valid and reliable (cups of FV estimated by the screener were moderately associated with intake measured via multiple 24-h recalls, and the 2–3-week test-retest correlation for FV intake was strong) [38]. To facilitate comparisons with a previous study relating self-reported FV intake to VM scores [12], two FV intake groups were created based on the median split of the distribution of responses: consuming less than three cups of FV/day and consuming three or more cups of FV/day.

### 2.3. Statistical Analysis

Descriptive statistics were used to characterize VM scores, participant characteristics, and self-reported FV intake at baseline. The 3- and 6-month test-retest reliability of skin carotenoid measurements was examined among the 137 participants in the usual care control study group with Pearson correlations (calculated from baseline to mid- and post-intervention, respectively). Relationships between the scores, participant characteristics, and self-reported FV intake were examined at baseline with Pearson correlations, independent samples *t* tests, and one-way analysis of variance with Bonferroni or Games-Howell post hoc tests as appropriate. VM scores were then regressed on the linear combination of variables found to be significantly associated with the scores in bivariate analyses. Two analyses were conducted. The first included potential determinants assessed in the full sample of participants. In the second analysis, measures of Hispanic origin (assessed among Hispanic participants only) were added (responses were dummy coded for the five Hispanic origin groups; “Other Hispanic” was the reference group). Post hoc power analyses revealed that for a prediction equation with nine predictors (the number in the second analysis) a minimum of 98 participants was needed to detect a moderate effect size of 0.15 with power of 0.80 [40]. Analyses were conducted with SPSS version 27 (IBM Inc., Armonk, NY, USA). Across analyses, a *p* value < 0.05 indicated statistical significance. 

## 3. Results

### 3.1. Participant Characteristics and VM Scores

The sample is described in detail elsewhere [31]. In brief, participants were primarily Hispanic (73%; most of Dominican origin), foreign-born (70%; most [78%] preferred to speak a language other than English [primarily Spanish]), with a mean age of 31.7 ± 7.2 years (Table S1). Half (55%) of participants reported a high school diploma or less; 18% and 25% were pregnant and breastfeeding, respectively, and 73% were overweight or obese (BMI > 25). The mean VM score in the sample was 270 ± 96 (range 0–695). A score of zero indicates that there is no detectable carotenoid absorption, i.e., potentially existing tissue carotenoid content is below the detection limit.

### 3.2. Repeatability of VM Scores

The 3-month test-retest correlation for skin carotenoid measurements was r = 0.79 (*p* < 0.001). The 6-month test-retest correlation was r = 0.55 (*p* < 0.001). Correlations of this magnitude indicate a strong association between measurements [41].

### 3.3. Bivariate Relationships with VM Scores

VM scores differed by Hispanic origin and were higher among participants of Ecuadorian (296 ± 127), Dominican (295 ± 89), and Mexican (298 ± 104) origin as compared to those of Puerto Rican (210 ± 61) origin (Table S1). VM scores also differed by nativity, breastfeeding and smoking status, and self-reported FV intake. The scores were higher among foreign-born (290 ± 102) than US-born participants (222 ± 60), participants who were breastfeeding (293 ± 93) relative to those who were not breastfeeding (262 ± 96), nonsmokers (272 ± 96) as compared to smokers (208 ± 67), and participants who consumed three or more cups of FV/day (282 ± 105) relative to those who consumed less than three cups of FV/day (253 ± 82). VM scores were negatively associated with BMI (r = −0.22, *p* < 0.001) and were lower among participants who were obese (244 ± 78) relative to those who were overweight (284 ± 102) and underweight and normal weight (285 ± 104). 

### 3.4. Determinants of VM Scores

Results of the regression analyses are shown in Table 2. The model containing potential determinants assessed in the full sample of participants (nativity, breastfeeding status, smoking status, BMI, and self-reported FV intake) was significant (F_5, 281_ = 11.77, *p* < 0.001; *R^2^* = 0.17; adjusted *R^2^* = 0.16). Beta weights were significant for the following three variables: nativity (β = 0.28, *p* < 0.001), self-reported FV intake (β = 0.17, *p* = 0.002), and BMI (β = −0.15, *p* = 0.006). VM scores increased as BMI decreased and were higher among foreign- as compared to US-born participants and participants who consumed three or more cups of FV/day relative those who consumed less than three cups of FV/day. The second model (in which measures of Hispanic origin were added) was also significant (F_9, 198_ = 6.16, *p* < 0.001; *R*^2^ = 0.22, adjusted *R*^2^ = 0.18). Among Hispanics, the same relationships were observed: VM scores increasing as BMI decreased and higher scores found among foreign- as compared to US-born participants and participants who consumed three or more cups of FV/day relative those who consumed less than three cups of FV/day.

## 4. Discussion

In this sample of urban, WIC-enrolled adults, VM scores ranged from 0–695 (mean score = 270 ± 96). The scores differed by Hispanic origin, nativity, breastfeeding, smoking, and weight status; and self-reported FV intake, and were negatively associated with BMI. In multivariate analyses, foreign-born nativity, consumption of three or more cups of FV/day, and smaller body size were determinants of increased VM scores.

The mean VM score of 270 in the present sample was comparable to the mean score of 284 found elsewhere among urban WIC-enrolled adults but was higher than the mean score of 175 found among low-income adults receiving aid from food assistance agencies (Table 1) [11,13]. Most (80%) of the 175 low-income adults were food insecure and were receiving FV through faith congregations partnering with After the Harvest, a nonprofit agency that rescues FV from going to waste and donates them to collaborating agencies [42]. Whereas WIC provides monthly cash value vouchers for FV (valued at $11 for women and $9 for children in New Jersey) [43], the frequency with which FV were provided to the 175 adults through the After the Harvest program is unclear. If infrequent, this may explain the lower mean VM score in this group. The difference between scores may also be an artifact of other differences between the samples. For example, whereas in the present study, participants were young mothers who were on average 31 years old, the sample of 175 adults was primarily female with a mean age of 53 years, and was comprised primarily of retirees, homemakers, and the unemployed [13]. The mean score of 270 was also comparable to the mean score of 299 found among primarily Hispanic, low-income women residing in colonias, unincorporated impoverished settlements with substandard living conditions, along the US-Mexico border (Table 1) [44]. The colonias residents were recruited at a health fair and were part of an outreach initiative to increase FV consumption, possibly explaining why the score in the colonias sample was similar to the score in the present sample. The mean score of 270 in the present sample was lower than the mean score of 342 found in a sample of socioeconomically diverse adults (Table 1) [10]. This is not surprising considering that FV intake increases with income [45,46].

Skin carotenoid status as measured by an alternate optical method, resonance Raman spectroscopy, is highly repeatable over 6 months [7,47]. The present findings aid understanding of the repeatability of skin carotenoid status as measured by the VM. The strong 3- and 6-month test-retest correlations found demonstrate that carotenoid levels as measured by the VM are also stable over time.

For some potential predictors of VM scores, direct comparisons with previous findings are not possible owing to a paucity of studies. However, indirect comparisons with studies of self-reported FV intake may be meaningful. The lower VM score among Puerto Rican as compared to Ecuadorian, Dominican, and Mexican participants is consistent with research demonstrating that of Cuban, Mexican, Dominican, Puerto Rican, Central American, and South American adults, those of Puerto Rican origin had the lowest FV intakes [48]. In the investigators’ previous work with adults served by the collaborating WIC agency, foreign-born Hispanic participants had higher intakes of orange-colored vegetables (FV high in carotenoids) than their US-born counterparts, possibly explaining the higher VM scores among the primarily foreign-born Hispanic participants in this study [26,49]. Most foreign-born participants in this study preferred to speak a language other than English, suggesting that they were less acculturated than foreign-born participants preferring to speak English. The higher VM score among foreign-born participants may therefore be an artifact of acculturation, shown to vary inversely with FV intake [50,51]. The homogeneity of language preference among foreign-born participants may explain why FV intake did not differ by language preference in this group. 

The higher VM score among breastfeeding women is consistent with research demonstrating that in low-income samples, FV intake is higher among breastfeeding than non-breastfeeding women [52,53]. The higher FV intake may be explained by maternal beliefs about the importance of a healthy diet. In focus group discussions with low-income women who chose to breastfeed, respondents saw a connection between maternal diet and infant health and were motivated to eat healthfully to benefit the child [54]. 

Considering that each 100 units of the VM score corresponds to consumption of one cup of FV/day, the mean VM score in the present sample translates to consumption of 2.7 cups of FV/day. This is well below the recommended 3.5 to 5 cups of FV/day [55], highlighting the need for dietary intervention programs and policies to promote FV intake in the WIC population. As found elsewhere, the scores distinguished participants who consumed three or more cups of FV/day from those who consumed less than three cups/day, suggesting that the VM may be useful for ranking individuals who consume high and low levels of FV [12].

The higher VM score among nonsmokers is consistent with research demonstrating that smokers have lower levels of skin carotenoids than non-smokers [56]. This is likely due to the direct effects of tobacco-induced oxidation of carotenoids and to lower intake of carotenoids [56]. The negative association with BMI is also consistent with previous findings [10,16,17]. Research has shown that low carotenoid levels are a risk factor for overweight and obesity; as such, a low VM score may be a determinant of obesity [57]. It has also been suggested that low carotenoid levels may be the result of abnormal weight gain as adipose tissue generates oxidative stress and in defending against the stress, carotenoid levels are reduced [58]. A low VM score may therefore be a consequence of excess weight gain. The present findings add to this work.

Across analyses, foreign-born nativity, consumption of three or more cups of FV/day, and smaller body size were predictors of increased VM scores. Investigators seeking to profile VM scores in primarily Hispanic, urban WIC-enrolled adults are therefore encouraged to assess these determinants and to adjust analyses of the scores for their potentially confounding effects. Moreover, interventions to promote FV intake in this population should be designed to target those most at risk of low VM scores and correspondingly, low FV intake, i.e., adults who are US-born, an unhealthy weight, and report consuming low levels of FV. Further research is needed to confirm these findings and to identify other demographic and psychosocial determinants of VM scores. Warranting examination are psychosocial variables consistently shown to influence adult FV intake as measured by self-report, e.g., FV access, availability, and taste preferences [9,10,11,12,13,14,15,16,17,18,19,20,21,22,23,24,25,26,27,28,29,30,31,32,33,34,35,36,37,38,39,40,41,42,43,44,45,46,47,48,49,50,51,52,53,54,55,56,57,58,59,60,61].

### Study Limitations and Strengths

When assessing skin carotenoid levels, we did not scan the same hand (dominant or nondominant), which may have affected the reliability of measurements. VM scores have been shown to vary by hand and digit [62]. In longitudinal studies of this type, investigators assessing skin carotenoids with the VM are therefore encouraged to scan the same finger at each assessment. Measurements may also have been affected by ambient temperature, which can change the blood flow in hand fingers (temperature-induced vasodilation or vasoconstriction). We considered this unlikely, however, as the VM measures the tissue in a relatively stable state where most blood is pushed away from the measured tissue volume. Moreover, the instrument’s spectral deconvolution algorithm corrects for any remaining oxy- and deoxyhemoglobin chromophores. Some participants had a VM score of zero. It is likely that participants with low dermal carotenoid levels also had low macular pigment levels and potentially resulting vision disorders [63,64]. Validating low scores, for example, by assessing eye function or screening for vision disorders, is therefore recommended. The approach used to estimate pre-pregnancy BMI among pregnant participants may have biased study findings. This was unlikely however, as all analyses were conducted twice (once with the estimated values and a second time excluding pregnant participants) and the results were the same, i.e., BMI was inversely related to VM scores in bi- and multivariate analyses. The total variance explained by the models predicting VM scores was small (0.17–0.22), suggesting that other factors not measured in this study are important for understanding variation in the scores. A brief screener was used to assess self-reported FV intake owing to concerns that a lengthier questionnaire combined with measures of targeted intermediate and secondary outcomes would be overly burdensome to participants. A disadvantage was that the screener did not capture intake of specific foods; as such, it was not possible to distinguish total FV intake from carotenoid-rich FV intake, shown to be associated with VM scores [8]. In future studies, the use of more comprehensive measures that allow for the assessment of a variety of FV including foods that are significant sources of carotenoids is therefore recommended.

Despite these limitations, this is one of the few studies to date to profile VM scores among low-income adults served by the WIC program. The study included a more comprehensive set of demographic variables than has been studied previously and is the first of which the authors are aware to examine the scores by Hispanic ethnicity and origin. This is also the first study to report VM scores observed in other adult samples, information that may aid understanding of how the scores in a particular sample compare with those found elsewhere.

## 5. Conclusions

Findings of this study aid understanding of VM scores among urban, primarily Hispanic low-income adults served by the WIC program and may provide a frame of reference for interpreting the scores in other low-income samples. Despite several statistically significant bivariate relationships between the scores, participant demographic characteristics, and self-reported FV intake, foreign-born nativity, consumption of three or more cups of FV/day, and smaller body size were determinants of increased VM scores. Investigators working with similar populations are therefore encouraged to target these determinants in observational and intervention studies of FV intake as measured by the VM.

## Figures and Tables

**Table 1:** Veggie Meter scores in adult samples.

Reference	Sample (Sex, Race/Ethnicity, and Age)	Mean Score	Range
Rush et al. [10]	571 adults from diverse community groups in New Zealand (57% female; 7% Māori, 15% Pacific, 17% Asian, 13% South Asian, 41% European, and 8 Other; mean age 39 ± 17 years (range 16–85 years))	342 ± 116 315 ± 110 (Māori) 257 ± 85 (Pacific) 388 ± 115 (Asian) 341 ± 113 (South Asian) 352 ± 111 (European) 367 ± 126 (Other)	83–769
McGuirt et al. [11]	136 adults recruited through two North Carolina supermarkets (76% female; 86% AA; mean age 46 ± 15 years)	251 ± 75 284 (WIC-enrolled) 247 (not WIC-enrolled)	11–450
Hill et al. [12]	84 Alaska Native adults (45% male; 84% Yup’ik Alaska Native; mean age 48 years)	222 ± 106	NR
Valentine et al. [13]	57 adults receiving aid from food pantries and food assistance agencies in the Kansas City area	175 ± 77	NR
Stephenson et al. [14]	525 farmers’ market patrons in Kentucky	200 ± 88	NR
Jilcott Pitts et al. [15]	479 corner store customers in North Carolina (41% female; 66% AA; mean age 43 ± 15 years)	234 ± 86	NR
Pitts et al. [8]	30 adults in Eastern North Carolina recruited from a medical school email listserv (57% AA; mean age 33 ± 12 years)	296 ± 110	NR
Jones et al. [16]	40 undergraduate and graduate students at a public university in California (72% female)	334	NR
Obana et al. [17]	985 patients and staff members of an ophthalmology clinic in Hamamatsu Seirei Hospital, Japan (59% male; mean age 70 ± 14 years)	343 ± 142	32–914
Keller at al. [18]	61 cognitively normal adults ≥ 65 years of age participating in a nutrition intervention at the University of Kansas Medical Center	279 ± 72	NR
Ermakov et al. [9]	54 adults from a diverse cohort of patients and staff at a tertiary care eye clinic in Utah	297	NR
	569 adults enrolled in a clinical epidemiology study carried out at the eye clinic of the Hamamatsu Seirei Hospital, Japan	335	32–892
	49 adults residing in colonias along the US-Mexico border (77% female; 90% Hispanic)	299	NR
Jilcott Pitts et al. [19]	279 adults participating in a policy intervention to promote healthier foods among small food retailers in North Carolina (Int: 44% female; 40% AA; mean age 44 ± 15 years; Ctl: 42% female; 87% AA; mean age 44 ± 14 years)	230 ± 72 (Int) 241 ± 100 (Ctl)	NR
Thompson et al. [20]	26 federal government employees participating in an online dietary intervention (54% male; 89% non-Hispanic white)	229	NR
Kelley et al. [21]	649 farmers’ market shoppers (381 in NYC and 268 in rural NC) (80% female; 45% Caucasian; 37% aged 60+ years)	290 315 (NYC shoppers) 253 (NC shoppers)	NR

AA: African American; WIC: Special Supplemental Nutrition Program for Women, Infants and Children; NR: not reported; Int: intervention group; Ctl: control group; NYC: New York City; NC: North Carolina.

**Table 2:** Regression analyses of participant characteristics and self-reported fruit and vegetable intake predicting Veggie Meter scores.

	Model 1		Model 2	
	Full Sample (*n* = 297)		Hispanics (*n* = 217)	
Predictor	β	SE	β	SE
Nativity	0.28 ***	11.7	0.20 **	18.2
Breastfeeding status	0.09	12.3	0.06	14.5
Smoking status	−0.10	23.3	−0.10	36.1
BMI	−0.15 **	0.80	−0.18 **	1.0
Self-reported FV intake	0.17 ***	10.6	0.20 **	12.5
	Model *R*^2^ = 0.17			
Puerto Rican origin			−0.10	23.7
Ecuadorian origin			0.14	20.9
Dominican origin			0.10	17.0
Mexican origin			0.11	21.2
			Model *R*^2^ = 0.22	

** *p* < 0.01; *** *p* < 0.001. β: standardized regression coefficient; SE: standard error; FV: fruits and vegetables; R^2^: coefficient of determination. Categorical variables were dummy coded (0,1) as follows: nativity (foreign-born = 1), breastfeeding status (breastfeeding = 1), smoking status (being a smoker = 1), and self-reported FV intake (consumption ≥ 3 cups/day = 1); for each Hispanic origin group, membership in the group = 1.

## Data Availability

The data presented in this study are available on request from the corresponding author.

## Supplementary Materials

The following are available online at https://www.mdpi.com/article/10.3390/nu13072270/s1, Table S1: Participant characteristics and self-reported fruit and vegetable intake and bivariate relationships with Veggie Meter scores.
