# Supplementary material for: Use of the Spectroscopy-Based Veggie Meter® to Objectively Assess Fruit and Vegetable Intake in Low-Income Adults
Source: Nutrients. 2021 Jun 30;13(7):2270. doi: 10.3390/nu13072270 (PMC8308249; doi:10.3390/nu13072270)
Supplement: Supplementary file 1 [file nutrients-13-02270-s001.zip › nutrients-1248460-supplementary.pdf]

Table S1. Participant characteristics and self-reported fruit and vegetable intake and bivariate relationships with Veggie Meter scores.

Characteristic	Number (%) or Mean ± SD	Pearson correlation or Mean VM Score ± SD
Age, mean ± SD	31.7 ± 7.2	0.04
	Race/ethnicity	
Hispanic	217 (73%)	276 ± 98
Non-Hispanic Black/African American	37 (13%)	259 ± 98
Non-Hispanic White or other	43 (14%)	245 ± 82
	Hispanic origin (Hispanic)***	
Puerto Rican	27 (12%)	210 ± 61[a]
Ecuadorian	32 (15%)	296 ± 127[b]
Dominican	73 (34%)	295 ± 89[b]
Mexican	30 (14%)	298 ± 104[b]
Other Hispanic	55 (25%)	260 ± 86[a,b]
	Nativity***	
US-born	88 (30%)	222 ± 60
Foreign-born	209 (70%)	290 ± 102
Years in US (foreign-born), mean ± SD	10.9 ± 7.6	-0.05
	Preferred language (foreign-born)	
English	46 (22%)	291 ± 98
Other than English	163 (78%)	289 ± 103
	Educational attainment	
Some high school or less	61 (20%)	258 ± 106
High school diploma or equivalent	103 (35%)	258 ± 97
More than high school	133 (45%)	284 ± 90
	Pregnancy status	
Not pregnant	243 (82%)	275 ± 97
Pregnant	54 (18%)	246 ± 93
	Breastfeeding status*	
Not breastfeeding	224 (75%)	262 ± 96
Breastfeeding	73 (25%)	293 ± 93
	Car ownership	
Did not own a reliable car	122 (41%)	273 ± 97
Owned a reliable car	175 (59%)	267 ± 96
	Car access	
Poor	71 (24%)	283 ± 85
Fair	41 (14%)	281 ± 113
Good	37 (12%)	284 ± 84
Very good	41 (14%)	257 ± 109
Excellent	107 (36%)	256 ± 95
	Food security status	
Food insecure	118 (40%)	271 ± 75
Food secure	174 (60%)	268 ± 108
	Smoking status*	
Nonsmoker	274 (95%)	272 ± 96
Smoker	16 (5%)	208 ± 67
	Past 7-day exposure to secondhand smoke	
No	251 (85%)	273 ± 98
Yes	44 (15%)	252 ± 85

	Supplement use	
Not taking supplements	145 (49%)	265 ± 86
Taking supplements	152 (51%)	274 ± 105
Body mass index, mean ± SD***	29.0 ± 6.9	-0.22
	Weight status category**	
Underweight and normal weight	81 (27%)	285 ± 104$_a$
Overweight	105 (36%)	284 ± 102$_a$
Obese	110 (37%)	244 ± 78$_b$
	Meeting physical activity guidelines	
No	123 (41%)	275 ± 94
Yes	174 (59%)	266 ± 98
	Self-reported FV intake (cups of FV/day)**	
< 3 cups/day	129 (44%)	253 ± 82
⩾ 3 cups/day	167 (56%)	282 ± 105

*p < 0.05; **p < 0.01; ***p < 0.001. Means with differing subscript letters differ significantly (p < .05). VM: Veggie Meter; SD: standard deviation; FV: fruits and vegetables. Sample sizes varied due to missing data for some measurements.
